# Heparin-binding protein (HBP) improves prediction of sepsis-related acute kidney injury

**DOI:** 10.1186/s13613-017-0330-1

**Published:** 2017-10-18

**Authors:** Jonas Tverring, Suvi T. Vaara, Jane Fisher, Meri Poukkanen, Ville Pettilä, Adam Linder, the FINNAKI Study Group

**Affiliations:** 1 Division of Infection Medicine, Department of Clinical Sciences, Lund University, BMC B14, 221 84 Lund, Sweden; 2 Division of Intensive Care Medicine, Department of Anesthesiology, Intensive Care and Pain Medicine, University of Helsinki and Helsinki University Hospital, Helsinki, Finland; 3 Department of Intensive Care, Austin Hospital, Melbourne, Australia; 4 Department of Intensive Care, Lapland Central Hospital, Rovaniemi, Finland

**Keywords:** Acute kidney injury, Sepsis, Biomarker, Heparin-binding protein, Risk model

## Abstract

**Background:**

Sepsis-related acute kidney injury (AKI) accounts for major morbidity and mortality among the critically ill. Heparin-binding protein (HBP) is a promising biomarker in predicting development and prognosis of severe sepsis and septic shock that has recently been proposed to be involved in the pathophysiology of AKI. The objective of this study was to investigate the added predictive value of measuring plasma HBP on admission to the intensive care unit (ICU) regarding the development of septic AKI.

**Methods:**

We included 601 patients with severe sepsis or septic shock from the prospective, observational FINNAKI study conducted in seventeen Finnish ICUs during a 5-month period (1 September 2011–1 February 2012). The main outcome measure was the development of KDIGO AKI stages 2–3 from 12 h after admission up to 5 days. Statistical analysis for the primary endpoint included construction of a clinical risk model, area under the receiver operating curve (ROC area), category-free net reclassification index (cfNRI) and integrated discrimination improvement (IDI) with 95% confidence intervals (95% CI).

**Results:**

Out of 511 eligible patients, 101 (20%) reached the primary endpoint. The addition of plasma HBP to a clinical risk model significantly increased ROC area (0.82 vs. 0.78, *p* = 0.03) and risk classification scores: cfNRI 62.0% (95% CI 40.5–82.4%) and IDI 0.053 (95% CI 0.029–0.075).

**Conclusions:**

Plasma HBP adds predictive value to known clinical risk factors in septic AKI. Further studies are warranted to compare the predictive performance of plasma HBP to other novel AKI biomarkers.

**Electronic supplementary material:**

The online version of this article (doi:10.1186/s13613-017-0330-1) contains supplementary material, which is available to authorized users.

## Background

Acute kidney injury (AKI) accounts for major morbidity and mortality among the critically ill, and septic shock is a leading cause for AKI [1]. Current AKI diagnosis is defined by a decrease in urine output (UOP) or an increase in serum creatinine (SCr) [2]. Unfortunately, SCr is a late marker of declined kidney function and does not adequately reflect damage to kidney cells. There is a broad consensus that more sensitive and specific biomarkers are needed [3]. Despite considerable research efforts, no novel biomarkers of AKI are currently in widespread clinical use. Heparin-binding protein (HBP), also known as azurocidin or cationic antimicrobial protein of 37 kDa, is a mediator of inflammation and vascular permeability that is released from activated neutrophils and has been shown to correlate with sepsis development, severity and prognosis [4–6]. HBP has recently been suggested to be involved in the pathophysiology of AKI with data from a murine model and a human cell line [7]. In two recent papers, plasma HBP performed well in predicting septic AKI, first among 296 patients with septic shock and second among 59 patients with severe sepsis, respectively [7, 8]. However, neither of those studies presented any statistical analysis on added predictive value to clinical risk factors. Accordingly, we aimed to investigate whether plasma HBP would add predictive value to known clinical risk factors regarding development of septic AKI.

## Methods

### Patients

This was a post hoc study of the prospective, observational, multicentre FINNAKI study. Patients with severe sepsis or septic shock diagnosed on day one and who had plasma samples available from admission to the ICU were included in the current study. The FINNAKI study consecutively included all emergency ICU admission and the elective admissions with an ICU stay of above 24 h from seventeen Finnish ICUs during a 5-month period (1 September 2011–1 February 2012) and reported the incidence, risk factors and 90-day mortality of patients with AKI. In brief, exclusion criteria for the FINNAKI study were patients who (1) had end-stage renal disease requiring maintenance dialysis, (2) were organ donors, (3) received intermediate care, (4) had received renal replacement therapy (RRT) while enrolled in the study during a previous ICU admission, (5) were transferred from another ICU where the data collection for the study was fulfilled or (6) were not permanently living in Finland or were unable to give consent due to insufficient language skills. Further details for the FINNAKI study have been published in detail previously [9].

### Definitions

AKI was defined and staged using the kidney disease: improving global outcomes (KDIGO) guidelines using both daily serum creatinine and hourly urine output measurements [2]. Baseline SCr was defined as the latest value from the previous year excluding the last week preceding admission. If baseline SCr was not available, SCr was estimated by the modification in diet in renal disease (MDRD) [10] equation assuming a glomerular filtration rate of 75 ml/1.73 m^2^. Severe sepsis and septic shock were defined using American College of Chest Physicians/Society of Critical Care Medicine guidelines [11].

### Data and sample collection

The Ethics Committee of the Department of Surgery, Helsinki and Uusimaa Hospital District approved the study protocol, and each participant or his/her proxy gave written informed consent. Patient demographics, medical history, severity scores, length of stay, physiologic data and hospital mortality were collected from the Finnish Intensive Care Consortium prospective database (Tieto Ltd, Helsinki, Finland) and with a study-specific case report form. AKI status was screened at admission and during the first 5 days of ICU stay. All data collection was blinded to the index test results. Plasma samples were collected immediately at ICU admission or after 2 h at the latest and directly centrifuged, aliquoted and frozen to − 80 °C. Plasma samples were sent on dry ice between Helsinki, Finland, and Lund, Sweden, for plasma HBP analyses.

### HBP test analyses

Plasma HBP concentration was measured in duplicate using a commercial HBP ELISA (Axis-Shield Diagnostics, Dundee, UK) according to the manufacturer’s directions. Intra-test variability was controlled through repeated analyses when the coefficient of variation (%CV) was above 10%. Analyses were performed with positive and negative controls, by the same laboratory personnel and blinded to clinical outcomes.

### Clinical endpoints

The primary endpoint was the development of new AKI stages 2–3 from 12 h after admission up to 5 days. The endpoint also included patients who developed AKI stage 2 within 12 h and then worsened to stage 3 within the 5 days. Secondary endpoints assessed all patients from admission to the ICU and included fluid balance within 24 h, maximum sequential organ failure assessment (SOFA) score within 5 days, initiation of RRT within 5 days and 28-day mortality, respectively.

### Sample size

The primary endpoint analysis (*n* = 511) and the secondary endpoint analyses (*n* = 601) were performed on available patients, samples and data.

### Statistical analyses

We constructed a risk model using multivariable logistic regression and compared its predictive performance for the primary endpoint with and without addition of plasma HBP using area under the receiver operating curve (ROC area) and category-free net reclassification index (cfNRI) and integrated discrimination improvement (IDI). CfNRI and IDI are presented for events, non-events and totals with bootstrapped 95% confidence intervals (95% CI) based on 10,000 replications. Testing for the equality of the ROC area was done using an algorithm suggested by DeLong [12]. We also present positive likelihood ratio (LR+) for categorised plasma HBP and ROC area for continuous plasma HBP to predict the primary endpoint. Two-by-two contingency tables were used to calculate sensitivity, specificity, positive predictive value (PPV) and negative predictive value (NPV) for plasma HBP at a binary cut-off. Sensitivity analyses were performed with changes to the primary endpoint regarding outcome definition, missing data, competing risks and baseline imbalance, respectively, and tested for ROC area with 95% CI. Univariable logistic regression was used to calculate the odds ratio (OR) presented in the baseline characteristics. The secondary endpoints were evaluated using independent-sample *t* tests, Mann–Whitney *U* test, ROC area, Kaplan–Meier survival curve and log rank (Mantel–Cox) test, as appropriate. We used missing at random assumptions and performed complete case analysis, in all cases except for baseline creatinine, which was required for AKI diagnosis, and was estimated using the MDRD equation [10]. Results are presented with 95% CI whenever applicable. Medians are presented together with interquartile ranges (IQRs). For all analyses, except when constructing the risk model, two-sided p values less than 0.05 were considered statistically significant. The software used for statistical analysis was SPSS (SPSS version 24.0, IBM Corp., Chicago, USA) and STATA (STATA MP 14.2, StataCorp, Texas, USA).

## Results

### Patient characteristics

**Fig. 1:**
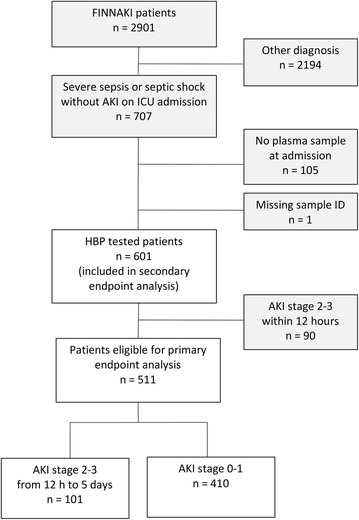
Flow chart of participants. HBP was tested on 601 patients, who were all included in the secondary endpoint analyses. Ninety patients were excluded from the primary endpoint analysis because they developed AKI stages 2–3 within 12 h, resulting in 511 patients eligible for the primary endpoint analysis

We included a total of 601 patients with severe sepsis or septic shock from the FINNAKI cohort and analysed HBP concentration on plasma samples from admission to the ICU. One patient was excluded due to missing identification number on target sample. Ninety patients were excluded from the primary endpoint analysis because they had already developed AKI stages 2–3 within 12 h, resulting in 511 evaluable patients in the primary analysis (Fig. 1). Baseline characteristics did not differ significantly regarding age, gender, baseline creatinine, source of admission, presence of hypertension, diabetes and medication pre-ICU admission except for use of colloid starch. However, patients developing stages 2–3 AKI had more often chronic kidney disease and positive blood cultures, as well as having a greater disease severity in terms of SAPS II score, vasopressor use on day one, development of septic shock, need for mechanical ventilation and higher creatinine and lactate levels pre-ICU admission, as compared to patients who did not develop stages 2–3 AKI (Table 1). For further data on infection characteristics, see Additional file 1; Table S1.

**Table 1:** Patient characteristics

	AKI stages 0–1 (*n* = 410, where of 127 stage 1)	AKI stages 2–3 (*n* = 101, where of 70 stage 3)	No data (*n*)	Odds ratio (95% CI) univariable	*p* value univariable	Odds ratio (95% CI) multivariable risk model
Age (years)	65 (54–74)	69 (56–79)	0	1.02 (1.00–1.03)	0.02	1.01 (1.00–1.03)
Gender (female)	153 (37.3%)	39 (38.6%)	0	1.06 (0.68–1.65	> 0.3	*
Weight (kg)	80 (68–90)	79 (68–91)	0	1.01 (1.00–1.02)	> 0.3	*
Baseline SCr (μmol/l)	75 (60–89)	83 (66–119)	118	1.01 (1.01–1.02)	< 0.001	^‡^
*Severity of disease*						
SAPS II score (points)	38 (30–46)	54 (40–64)	0	1.06 (1.05–1.08)	< 0.001	^§^
SAPS II without points for renal or age	23 (17–29)	26 (19–36)	0	1.03 (1.02–1.05)	< 0.001	1.04 (1.02–1.06)
Vasopressor on day one	265 (65%)	80 (79%)	0	2.08 (1.24–3.51)	< 0.01	^†^
Mechanical ventilation	253 (62%)	77 (76%)	0	1.99 (1.21–3.28)	< 0.01	^†^
Septic shock	289 (71%)	89 (88%)	0	3.11 (1.64–5.88)	< 0.01	^†^
*Comorbidity*						
Chronic kidney disease	24 (5.9%)	13 (13%)	0	2.38 (1.16–4.85)	0.02	^‡^
Renal transplant	5 (1.2%)	1 (1.2%)	2	0.81 (0.09–7.00)	> 0.3	*
Diabetes	93 (23%)	27 (27%)	0	1.24 (0.76–2.05)	> 0.3	*
Hypertension	211 (52%)	48 (48%)	2	0.85 (0.55–1.31)	> 0.3	*
Systolic heart failure	48 (12%)	9 (8.9%)	4	0.76 (0.36–1.61)	> 0.3	*
COPD	63 (15%)	12 (12%)	6	0.74 (0.38–1.43)	> 0.3	*
Any malignancy	53 (13%)	18 (18%)	0	1.46 (0.81–2.62)	0.21	^‡^
Chronic liver failure	17 (4.1%)	4 (4.0%)	7	0.96 (0.32–2.92)	> 0.3	*
*Source of admission*						
Emergency department	142 (35%)	34 (34%)	2	0.95 (0.60–1.51)	> 0.3	*
Hospital ward	136 (33%)	35 (35%)	2	1.06 (0.67–1.68)	> 0.3	*
Operating room	87 (21%)	27 (27%)	2	1.35 (0.82–2.22)	0.24	^‡^
High-dependency unit	28 (6.8%)	5 (5.0%)	2	0.71 (0.27–1.88)	> 0.3	*
Other	15 (3.7%)	0	2	0.00 (0.00–0.00)	> 0.3	*
*Laboratory results max pre-ICU*						
SCr (μmol/l) 48 h	85 (62–122)	156 (92–248)	21	1.01 (1.01–1.02)	< 0.001	1.01 (1.01–1.02)
Lactate (mmol/L) 24 h	2.0 (1.2–3.4)	3.3 (1.9–6.1)	184	1.14 (1.06–1.22)	< 0.001	^#^
Leucocyte (10^9/L) 24 h	12 (8–17)	14 (8–18)	50	1.01 (0.99–10.3)	> 0.3	*
CRP (mg/L) 24 h	157 (64–270)	176 (52–257)	17	1.00 (1.00–1.00)	> 0.3	*
*Treatment 48* *h pre-ICU*						
Immunosuppressive	36 (8.8%)	10 (9.9%)	5	1.13 (.054–2.35)	> 0.3	*
ACE inhibitor or ARB	107 (26%)	22 (22%)	9	0.82 (0.48–1.38)	> 0.3	*
NSAID	66 (16%)	15 (15%)	30	0.87 (0.47–1.60)	> 0.3	*
Diuretic	168 (41%)	44 (44%)	22	1.13 (0.72–1.78)	> 0.3	*
Colloid starch	48 (12%)	23 (23%)	17	2.19 (1.26–3.81)	< 0.01	^‡^
Radiocontrast	88 (22%)	21 (21%)	2	0.96 (0.56–1.63)	> 0.3	*
*Source of infection*						
Pulmonary	224 (55%)	34 (34%)	0	0.42 (0.27–0.67)	< 0.001	^†^
Abdominal	94 (23%)	32 (32%)	0	1.56 (0.97–2.52)	0.07	^†^
Urinary tract	20 (5%)	16 (16%)	0	3.67 (1.83–7.38)	< 0.001	^†^
Skin and soft tissue	33 (8%)	8 (8%)	0	0.98 (0.44–2.20)	> 0.3	*
*Microbiology*						
Blood culture positive	83 (20%)	31 (31%)	133	1.97 (1.16–3.35)	0.01	^†^
*E. coli*	11 (2.7%)	9 (8.9%)	133	3.76 (1.50–9.44)	< 0.01	^†^
Other gram negative	23 (5.6%)	8 (7.9%)	133	1.51 (0.65–3.53)	> 0.3	*
*Strep. pneumoniae*	14 (3.4%)	5 (5.0%)	133	1.53 (0.53–4.39)	> 0.3	*

Binary variables are shown as absolute number (percentage), and continuous variables are shown as median (interquartile range). Odds ratio, 95% CI and *p* values are calculated using univariable logistic regression towards the primary endpoint for the purpose of constructing a clinical risk model. Explanation for variable exclusion from the risk model follows

*SAPS* Simplified Acute Physiology Score; *COPD* chronic obstructive pulmonary disease; *CRP* C-reactive protein; *ACE* angiotensin-converting-enzyme inhibitor; *ARB* angiotensin II receptor blockers; *NSAID* non-steroidal anti-inflammatory drugs

* Excluded from the risk model due to *p* value above 0.3 in univariable logistic regression

^†^Excluded because the variable was not indisputably available to treating clinician at admission

^‡^Excluded due to *p* value above 0.1 in multivariable logistic regression

^§^Excluded because the risk model already contains SAPS without renal or age points

^#^Excluded due to too many missing values

### Patient outcomes

Out of 511 patients, 101 (20%) reached the primary endpoint of KDIGO AKI stages 2–3 from 12 h after admission up to 5 days. Thirty-one (6%) patients developed at highest AKI stage 2, and 70 (14%) patients developed at AKI stage 3, out of which 48 (9%) received renal replacement therapy (RRT). Four hundred and ten patients (80%) did not develop stages 2–3 AKI; 283 patients never developed AKI (55%); and 127 patients developed stage 1 (25%), as shown in Fig. 1. Data on fluid balance were available for 550 patients (8.5% missing) and were collected on the first day from ICU admission with a median time to measurement of 17 h (IQR 11–22 h). Median fluid balance in absolute volume was positive 1209 ml (IQR − 18 to + 3085 ml) and in fluid balance (kg) per weight was + 1.6% (IQR 0.0–4.1%). Median of maximum SOFA score during the first 5 days was 8 points (IQR 6–11) for all 601 patients. A total of 145 patients (24.1%) died within 28 days from ICU admission.

### HBP test results

Mean plasma HBP concentration on ICU admission for all patients was 40 ng/ml, and standard deviation was 65 ng/ml. Median plasma HBP was 19 ng/ml, and IQR was 9.1–39 ng/ml. The distribution of plasma HBP was left-shifted as compared to the normal curve. We set the binary cut-off for a high versus a low plasma HBP at 20 ng/ml, which is comparable to that of other studies (15 ng/ml in Linder et al. [5] and 30 ng/ml in Linder et al. [6]). Categorised plasma HBP based on quartiles resulted the following groups: HBP ≤ 10 ng/ml, HBP > 10 ≤ 20 ng/ml, HBP > 20 ≤ 40 ng/ml and HBP > 40 ng/ml, respectively. Among primary endpoint positive patients, plasma HBP was significantly elevated compared to endpoint negative patients’ plasma HBP (mean 59 ng/ml and median 33 ng/ml (IQR 15–73 ng/ml) versus mean 28 ng/ml and median 15 ng/ml (IQR 8–29 ng/ml), *p* < 0.001, *n* = 511). Among all patients (*n* = 601) reaching at highest AKI stages 0, 1, 2 and 3, median plasma HBP was 14 ng/ml (IQR 7–28 ng/ml), 19 ng/ml (IQR 9–37 ng/ml), 26 ng/ml (IQR 11–70 ng/ml) and 30 ng/ml (IQR 15–76 ng/ml), respectively. The plasma HBP levels for these groups differed significantly in individual comparison between groups in all cases except between AKI stage 2 versus 3, as shown in Additional file 2; Figure S1.

### Risk model construction

Only variables presented in Table 1 that were considered indisputably available to the treating physician at ICU admission were eligible for inclusion in the risk model. Variables that had a *p* value below 0.3 in a univariate logistic regression were included into a multivariable logistic regression and excluded one variable at a time, starting with the highest *p* value, until only variables with a *p* value below 0.1 remained. Established AKI risk factors with a *p* value above 0.3 (CKD, hypertension, diabetes and urinary tract infection) were not included in the risk model because their addition did not affect the ROC area or 95% CI at three decimal places. The final risk model included 489 patients and contained three variables with positive coefficients for the primary endpoint: age, SAPS II without renal and age points and maximum SCr 48 h pre-ICU admission. See Table 1 for further details. Plasma HBP was added to the risk model as a categorical variable based on quartiles because it produced a slightly higher ROC area (0.01 absolute difference) compared to adding plasma HBP as a continuous variable.

### Primary endpoint

The ROC area for the risk model including categorical plasma HBP to predict the primary endpoint was 0.82 (95% CI 0.77–0.87) compared to 0.78 (95% CI 0.73–0.84) for the risk model alone (*p* = 0.03, *n* = 489). The total cfNRI was positive 62% (95% CI 41–82%) and total IDI positive 0.053 (95% CI 0.03–0.08). LR+ was 2.73 (95% CI 2.00–3.71) for patients with a plasma HBP above 40 ng/ml and 0.43 (95% CI 0.26–0.71) for patients with a plasma HBP below 10 ng/ml, respectively. Continuous plasma HBP alone had a ROC area of 0.70 (95% CI 0.64–0.76) to predict the primary endpoint. We performed six sensitivity analyses based on ROC area and continuous plasma HBP. See Table 2 and Additional file 1; Tables S2–S5 for further results.

**Table 2:** Risk model comparison with and without plasma HBP (*n* = 489)

	Value	95% CI
ROC area: risk model only	0.784*	0.734–0.835
ROC area: risk model + plasma HBP	0.819*	0.770–0.868
cfNRI event	37.4%	18.6–55.1
cfNRI non-event	24.6%	15.2–34.0
cfNRI total	62.0%	40.5–82.4
IDI event	0.042	0.02–0.63
IDI non-event	0.011	0.003–0.019
IDI total	0.053	0.029–0.075

Events refer to the development of the primary endpoint. Categorised plasma HBP based on quartiles was used in the analysis

*cfNRI* category-free net reclassification index, *IDI* integrated discrimination improvement

* The difference in ROC area between the risk model with and without plasma HBP was statistically significant (*p* = 0.03)

### Secondary endpoints

**Fig. 2:**
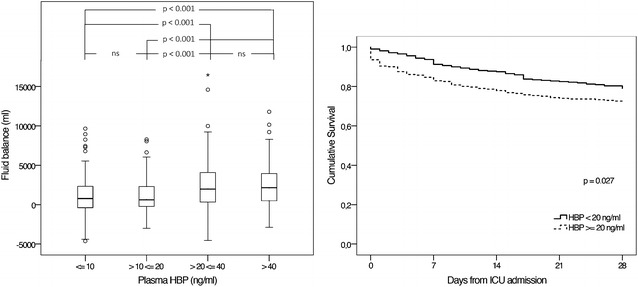
Fluid balance and 28-day survival. The left boxplot describes patients’ fluid balance within 24 h from ICU admission separated by plasma HBP quartiles and includes testing for significant difference between plasma HBP levels of each individual group (*n* = 601, *ns*: not significant). The right Kaplan–Meier survival curve pictures survival within 28 days from ICU admission among patients with a high versus low plasma HBP on ICU admission (*n* = 601)

Mean fluid balance within 24 h from ICU admission was significantly higher in patients with a high plasma HBP (≥ 20 ng/ml) compared to patients with a low plasma HBP on ICU admission (+ 2452 ml vs. + 1031 ml, *p* < 0.001). Mean difference in fluid balance per weight was 1.9% (95% CI 1.3–2.5%), and the mean difference in total volume was 1422 ml (95% CI 985–1859 ml). Median fluid balance for each defined plasma HBP quartile (≤ 10, > 10 ≤ 20, > 20 ≤ 40 and > 40 ng/ml) was 779 ml (IQR − 423 to 2337 ml), 612 ml (IQR − 246 to 2391 ml), 1978 ml (IQR 312–4086 ml) and 2146 ml (IQR 461–3995 ml), respectively, as shown in Fig. 2 and Additional file 3; Figure S2. Maximum SOFA score within 5 days from ICU admission was significantly higher in patients with a high plasma HBP compared to patients with a low plasma HBP on ICU admission (mean points 9.1 vs. 7.4, *p* < 0.001, *n* = 601) with a mean difference of 1.7 points (95% CI 1.2–2.2). Continuous plasma HBP alone had a ROC area of 0.69 (95% CI 0.63–0.75) to predict initiation of RRT for AKI within 5 days (*n* = 91 out of 601). Patients with a plasma HBP above 20 ng/ml on ICU admission had a significantly higher unadjusted 28-day mortality than patients with a low plasma HBP (28 vs. 21%, *p* = 0.03), as shown in Fig. 2.

## Discussion

### Key findings

We have found that plasma HBP measured on ICU admission improves prediction of sepsis-related AKI stages 2–3 among mixed general ICU patients. Furthermore, we found that patients with an increased plasma HBP (≥ 20 ng/ml) on ICU admission had a significantly higher fluid balance within 24 h from ICU admission, a higher maximum SOFA score within 5 days and an increased risk of dying within 28 days, respectively, as compared to patients with a low plasma HBP on ICU admission.

### Pathophysiological mechanism

**Fig. 3:**
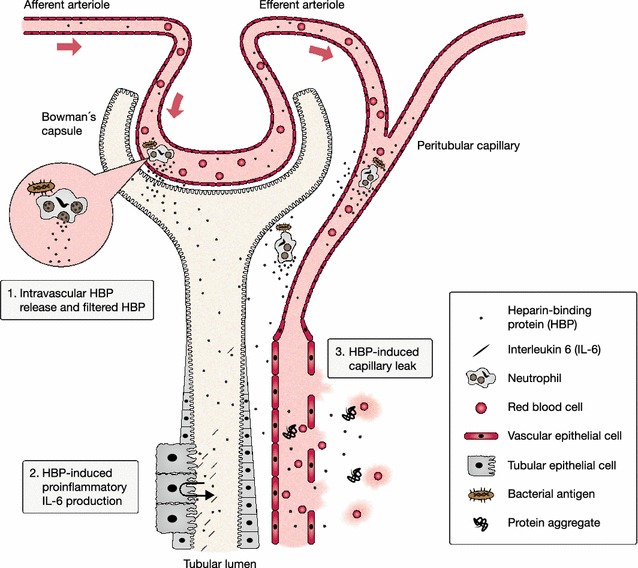
Proposed mechanism for HBP’s involvement in sepsis-related AKI pathophysiology. *1* Neutrophils activated by bacterial antigen release pre-produced HBP from secretory vesicles into peripheral tissue and blood vessels. HBP is filtered through the glomeruli, and the Bowman’s capsule into the tubular lumen *2* HBP induces inflammation in tubular epithelial cells, supported by evidence of IL-6 production [7]. *3* HBP act on peritubular vascular cells inducing capillary leakage through loosened tight junctions, supported by evidence of interstitial haemorrhage and protein aggregates in extracellular matrix [7]

HBP’s plausibility as a marker of septic organ dysfunction can be supported by its early release in response to an infection and its powerful effects on immune cells and endothelial cells, which may act as causative factors in sepsis [13]. Prefabricated HBP is rapidly released from secretory vesicles of activated neutrophils [14–16]. HBP act as a chemoattractant for neutrophils, T cells and monocytes and enhances monocyte cytokine release, phagocytosis and adhesion to the endothelium [17–20]. HBP also induces cytoskeletal rearrangement and cell contraction, forming gaps in the endothelium, leading to vascular leakage and neutrophil extravasation, which leads to more HBP release from azurophilic granules [21–23]. HBP also induces inflammation and capillary leakage in the kidney, as is supported by findings from Fisher et al. [7], which correspond to two out of three mechanisms in the proposed unifying theory of AKI pathophysiology presented by Gomez et al. [24]. On this background, we present a proposed mechanism to explain the findings from the primary endpoint analysis, as shown in Fig. 3.

### Primary endpoint interpretation and performance compared

Translating measures of diagnostic accuracy into clinical use is always a challenge. In this paper, we constructed a risk model to simulate the clinical information available to the treating clinician and then statistically measured the predictive benefit of adding the information from plasma HBP through cfNRI, IDI and improvement in ROC area. The cfNRI was 62%, indicating that about one-third of all patients will benefit from a more correct risk classification (cfNRI max is 200%) when adding plasma HBP to known clinical risk factors. The added predictive value is supported by a significant increase in ROC area of the risk model when adding plasma HBP. The risk model’s ROC area is comparable to that of previous studies (0.80 in Kashani et al. [25] and 0.86 in Honore et al. [26]).

We choose only to include patients who developed AKI beyond 12 h from admission in our primary analysis because we considered it most relevant to the treating clinician. AKI diagnosed within 12 h from sampling provides little time for possible clinical intervention. Furthermore, AKI diagnosed within 12 h will probably reflect kidney cell damage that was already present at biomarker sampling, due to the delayed nature of the current AKI definition based on SCr and UOP, arguably negating the predictive performance of the biomarker. This is probably also true in markers of cell cycle arrest, which are reasonably expressed when kidney cells are already distressed [25]. Conversely, there is evidence to suggest that plasma HBP may have a causative role in septic AKI [7], as shown in Fig. 3.

So far, only two previous studies have examined plasma HBP’s performance in predicting AKI. Fisher et al. [7] reported, first, a ROC area of 0.85 to discriminate patients with KDIGO AKI stage 0 versus 2 within 5 days from ICU admission, and second, a ROC area of 0.80 for discriminating AKI stage 0 versus 1–3 after 48 h up to 5 days from ICU admission among 296 patients with septic shock from the Vasopressin and Septic Shock Trial (VASST) cohort [27]. Tyden et al. [8] reported a ROC area of 0.70 for plasma HBP measured on ICU admission to predict the development of KDIGO AKI stages 2–3 within 7 days from ICU admission among 245 mixed ICU patients, and a ROC area of 0.88 for the same endpoint in a small subgroup analysis of 59 patients with severe sepsis. However, these results are not directly comparable to our results due to differences in primary endpoint definition, and there were no data on added benefit to clinical risk factors presented in either study.

Our results may be assessed in comparison with the cell cycle arrest biomarkers, namely urine tissue inhibitor of metalloproteinases-2 (TIMP-2) and insulin-like growth factor-binding protein 7 (IGFBP7). Honore et al. [26] present impressive results for the combined biomarker of urine [TIMP-2] * [IGFBP7] to predict KDIGO AKI stages 2–3 within 12 h in 232 ICU patients with severe sepsis and septic shock. However, these results do not clearly translate to our interest of AKI prediction beyond 12 h from admission. The original cohort of the above-mentioned study was published with a supplementary appendix [25] with results for the prediction of AKI (RIFLE Injury or Failure) diagnosed 12–36 h from sample collection in 522 septic and non-septic ICU patients. Here, the ROC area for urine [TIMP-2] * [IGFBP7] was 0.77, cfNRI was 70%, IDI was 0.098 and improvement in risk model ROC area just failed to be statistically significant (0.87 vs. 0.80, *p* = 0.06).

### Secondary endpoints interpretation

Patients with an increased plasma HBP on admission had a mean fluid balance that was 1422 ml higher than patients with a low plasma HBP within 24 h from ICU admission. This finding could arguably support HBP’s role as a marker of AKI since fluid overload is closely related to AKI and administration of RRT [28]. It also supports the biological role for HBP as a primary mediator of vascular leakage, an important pathophysiological mechanism in septic shock. A high plasma HBP was also associated with a higher maximum SOFA score within 5 days and an increased 28-day mortality, which is in line with earlier research correlating a high plasma HBP to greater sepsis severity and death [5].

### Limitations and strengths

First, the study was limited by being designed after sample and data collection. Second, samples had been stored at − 80 °C for over 1 year and shipped on dry ice from Finland to Sweden prior to being analysed. Third, we lacked baseline SCr for 30% of patients, where we had to back-calculate using the recommended MDRD formula. Fourth, no comparison to other biomarkers was made on the same patients and data, and there was no external validation performed. Strengths of this study include a biologically plausible biomarker, a generally accepted and clinically relevant endpoint, a large sample size from a well-characterised population and results that are robust to several types of statistical analyses.

## Conclusion

Plasma HBP is a biologically plausible novel biomarker of sepsis-related AKI that adds predictive value to known clinical risk factors. Further studies are warranted to compare the performance of plasma HBP to other novel AKI biomarkers.

## Additional files

**Additional file 1.** Heparin-binding protein (HBP) improves prediction of sepsis-related acute kidney injury.

**Additional file 2.** Boxplot comparing patient groups reaching their highest AKI stage from ICU admission up to five days, separated by plasma HBP quartiles. The figure includes testing for significant difference between plasma HBP levels of each individual group (n=601). ns: not significant.

**Additional file 3.** Scatter plot picturing each individual patient’s fluid balance within 24 hours from ICU admission correlated with his or her plasma HBP on ICU admission (n=601).

## Abbreviations

ACE: angiotensin-converting-enzyme inhibitor
AKI: acute kidney injury
ARB: angiotensin II receptor blockers
cfNRI: category-free net reclassification index
COPD: chronic obstructive pulmonary disease
CRP: C-reactive protein
%CV: coefficient of variation
ELISA: enzyme-linked immunosorbent assay
HBP: heparin-binding protein
ICU: intensive care unit
IDI: integrated discrimination improvement
IGFBP7: insulin-like growth factor-binding protein 7
IL-6: interleukin 6
LR+: positive likelihood ratio
MDRD: modification in diet in renal disease
ng/ml: nanograms per millilitre
NPV: negative predictive value
NSAID: non-steroidal anti-inflammatory drugs
OR: odds ratio
PASS: power analysis and sample size
PPV: positive predictive value
ROC area: area under the receiver operating curve
RRT: renal replacement therapy
SAPS: Simplified Acute Physiology Score
SCr: serum creatinine
SOFA score: sequential organ failure assessment
TIMP-2: tissue inhibitor of metalloproteinases-2
KDIGO: kidney disease: improving global outcomes
UOP: urine output
VASST: vasopressin and septic shock trial
