# Supplementary material for: Heparin-binding protein (HBP) improves prediction of sepsis-related acute kidney injury
Source: Ann Intensive Care. 2017 Oct 18;7:105. doi: 10.1186/s13613-017-0330-1 (PMC5647316; doi:10.1186/s13613-017-0330-1)
Supplement: Supplementary file 1 — Additional file 1. Heparin-binding protein (HBP) improves prediction of sepsis-related acute kidney injury. [file 13613_2017_330_MOESM1_ESM.docx]

Additional file 1

Heparin-binding protein (HBP) improves prediction of sepsis-related acute kidney injury

Jonas Tverring MD, Suvi T. Vaara MD PhD, Jane Fisher MSc, Meri Poukkanen MD PhD, Ville Pettilä MD PhD, Adam Linder MD PhD, the FINNAKI Study Group

| **Table 1.**  **Infection characteristics** | **AKI stage 0-1**  **(n = 410)** | **AKI stage 2-3**  **(n = 101)** | **Missing data (n)** | **P value** |
| --- | --- | --- | --- | --- |
| Taken blood culture | 296 (72%) | 74 (73%) | 14 | ns |
| Positive blood culture | 83 (20%) | 31 (31%) | 133 | 0.01 |
| Community acquired infection | 297 (72%) | 69 (68%) | 32 | ns |
| Nosocomial infection | 89 (22%) | 24 (22%) | 32 | ns |
| **Source of infection** |  |  |  |  |
| Pulmonary | 224 (55%) | 34 (34%) | 0 | <0.001 |
| Abdominal | 94 (23%) | 32 (32%) | 0 | ns |
| Urinary tract | 20 (5%) | 16 (16%) | 0 | <0.001 |
| Skin and soft tissue | 33 (8%) | 8 (8%) | 0 | ns |
| **Blood culture** |  |  |  |  |
| *E-coli* | 11 (2.7%) | 9 (8.9%) | 133 | <0.01 |
| Other gram negative * | 23 (5.6%) | 8 (7.9%) | 133 | ns |
| *Strep. pneumoniae* | 14 (3.4%) | 5 (5.0%) | 133 | ns |
| *Staph. aureus* | 10 (2.4%) | 1 (1.0%) | 133 | ns |
| Enterococcus | 5 (1.2%) | 2 (2.0%) | 133 | ns |
| Coagulase negative staph. | 11 (2.7%) | 0 (0%) | 133 | ns |
| Multiresistant bacteria † | 1 (0.2%) | 0 (0%) | 133 | ns |
| Candida spp. | 2 (0.5%) | 2 (2.0%) | 133 | ns |
| Other | 12 (2.9%) | 7 (6.9%) | 133 | ns |

*Data is shown as absolute number (percentage). P values were calculated using Fisher’s exact test. Ns indicates a p value above 0.05. * Haemophilus influenzae, Pseudomonas spp, Klebsiella spp, Bacteroides spp or Enterobacter spp.* † *Vancomycin-resistant enterococci, methicillin resistant staphylococcus aureus or extended spectrum beta lactamases.*

| **Table 2. Plasma HBP binary cut-off 20 ng/ml (n=511)** | | |
| --- | --- | --- |
|  | Value | 95% CI |
| Sensitivity | 76.2 % | 66.7 - 84.1 |
| Specificity | 48.4 % | 43.6 - 53.5 |
| Prevalence | 19.8 % | 16.4 - 23.5 |
| PPV | 26.7 % | 24.0 - 30.0 |
| NPV | 89.2 % | 85.2 - 92.3 |

*PPV: positive predictive value, NPV: negative predictive value*

| **Table 3.** | | | | |
| --- | --- | --- | --- | --- |
| **Plasma HBP categorical (n=511)** | **Positive likelihood ratio (LR+)** | | | |
|  | Value | 95% CI | n= |  |
| HBP < 10 ng/ml | 0.43 | 0.26 - 0.71 | 146 |  |
| HBP 10-20 ng/ml | 0.57 | 0.36 - 0.89 | 139 |  |
| HBP 20-40 ng/ml | 1.14 | 0.77 - 1.68 | 114 |  |
| HBP > 40 ng/ml | 2.73 | 2.00 - 3.71 | 112 |  |

*Categories were derived based on quartiles.*

| **Table 4. ROC area for continuous plasma HBP alone (n=511)** | |
| --- | --- |
| Value | 95% CI |
| 0.697 | 0.639-0.755 |

*ROC area: area under the Receiver Operating Curve*

| **Table 5. Sensitivity analyses** | | | | |
| --- | --- | --- | --- | --- |
| **Definition** | **ROC area** | **95% CI** | **n =** |  |
| Changed primary endpoint to start at 0 hours from admission instead of 12 hours | 0.681 | 0.635-0.727 | 601 |  |
| Excluding patients without baseline creatinine available | 0.657 | 0.582-0.731 | 361 |  |
| Excluding patients who received RRT | 0.688 | 0.612-0.764 | 458 |  |
| Excluding patients with leucocytes < 1 *10^9^/L at 24 hours before or after ICU admission | 0.709 | 0.646-0.771 | 440 |  |
| Including only blood culture positive patients | 0.717 | 0.609-0.825 | 114 |  |

*Sensitivity analyses are based on continuous plasma HBP alone on ICU admission to predict the primary endpoint.*

**Additional file 1 figure legends**

**Figure 1.** Boxplot comparing patient groups reaching their highest AKI stage from ICU admission up to five days, separated by plasma HBP quartiles. The figure includes testing for significant difference between plasma HBP levels of each individual group (n=601). ns: not significant.

**Figure 2.** Scatter plot picturing each individual patient’s fluid balance within 24 hours from ICU admission correlated to his or her plasma HBP on ICU admission (n=601).
