# Supplementary figures and images for: Heparin-binding protein (HBP) improves prediction of sepsis-related acute kidney injury
Source: Ann Intensive Care. 2017 Oct 18;7:105. doi: 10.1186/s13613-017-0330-1 (PMC5647316; doi:10.1186/s13613-017-0330-1)

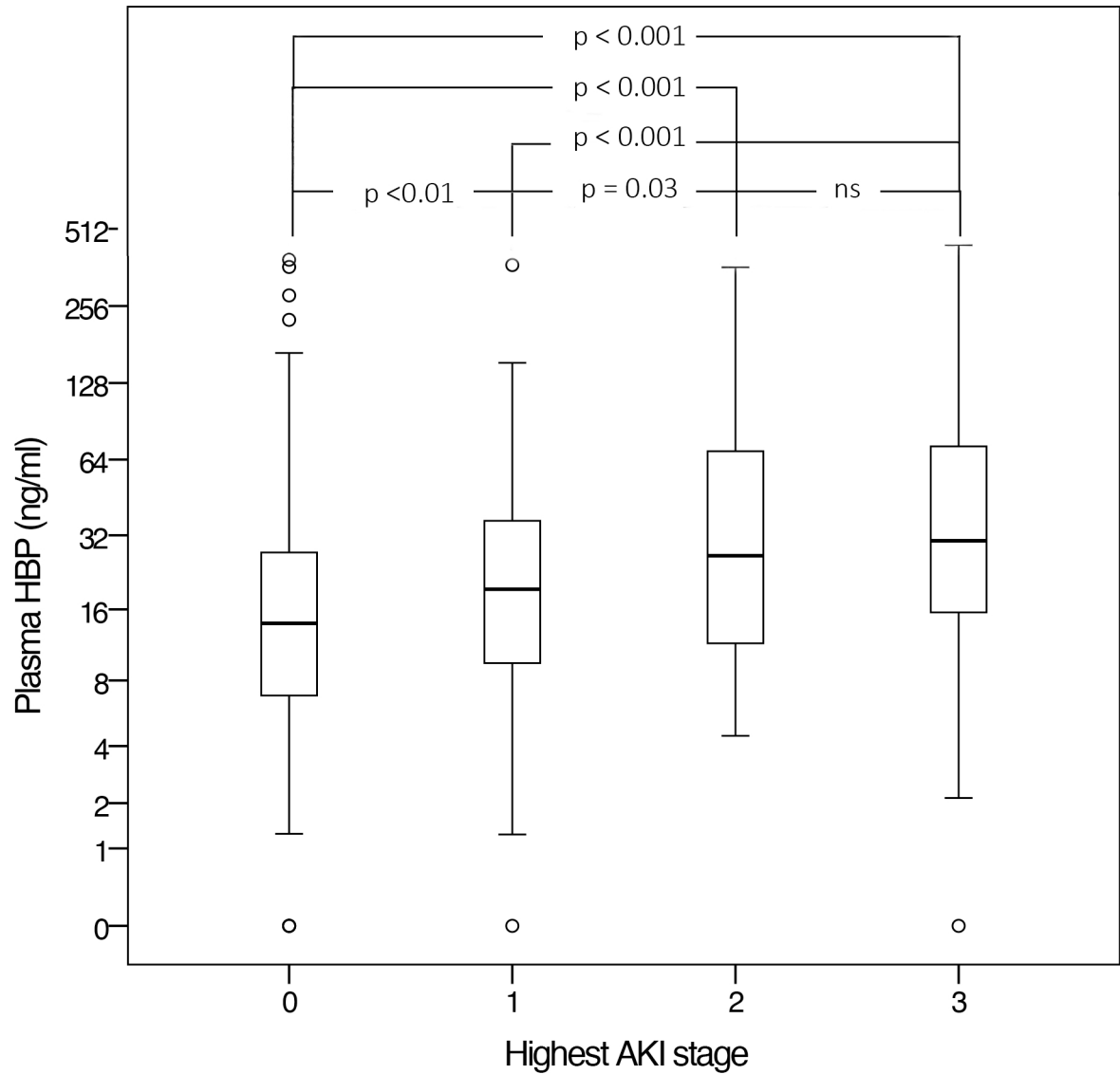

Supplement: Supplementary file 2 — Additional file 2. Boxplot comparing patient groups reaching their highest AKI stage from ICU admission up to five days, separated by plasma HBP quartiles. The figure includes testing for significant difference between plasma HBP levels of each individual group (n=601). ns: not significant. [file 13613_2017_330_MOESM2_ESM.pdf]

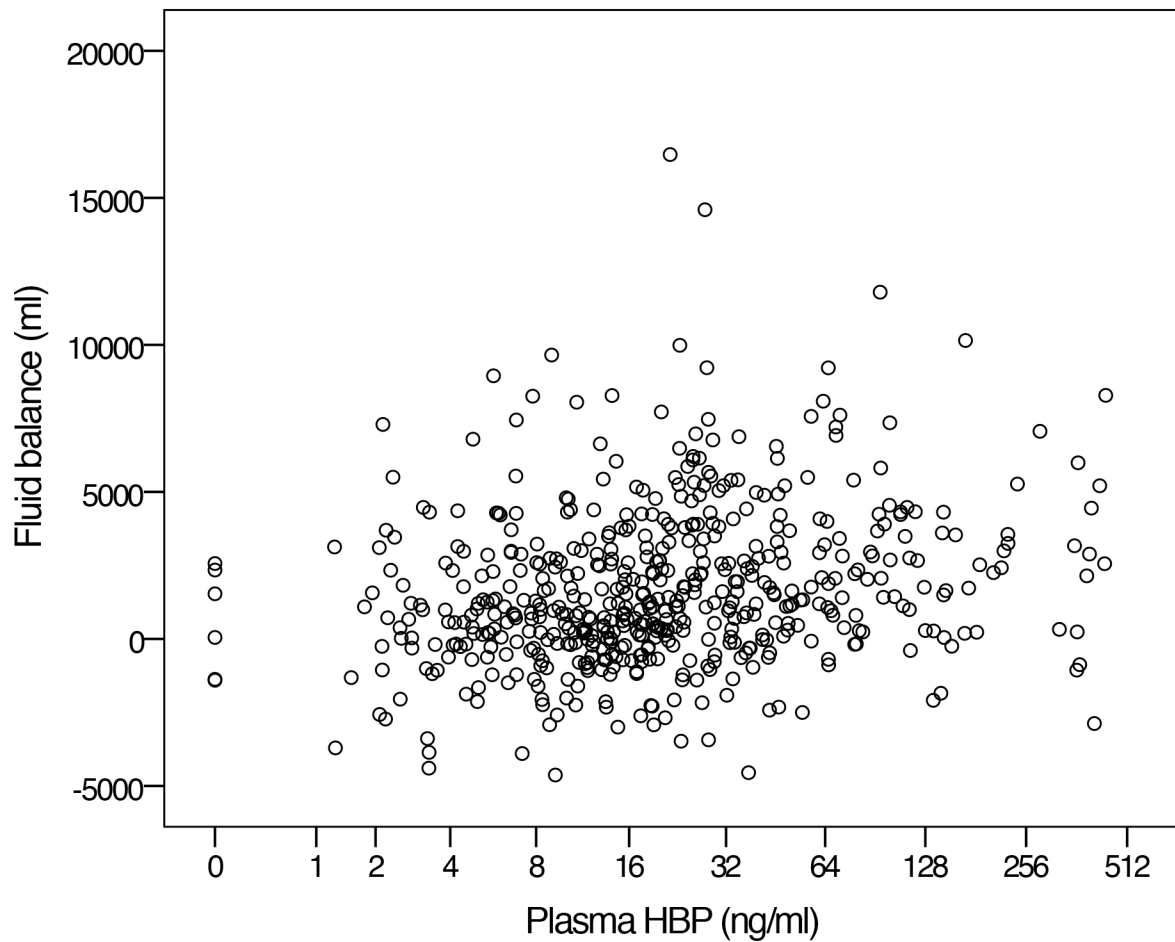

Supplement: Supplementary file 3 — Additional file 3. Scatter plot picturing each individual patient’s fluid balance within 24 hours from ICU admission correlated with his or her plasma HBP on ICU admission (n=601). [file 13613_2017_330_MOESM3_ESM.pdf]
